# Supplementary material for: “It just feels right”: Perceptions of the effects of community connectedness among trans individuals
Source: PLoS One. 2020 Oct 5;15(10):e0240295. doi: 10.1371/journal.pone.0240295 (PMC7535036; doi:10.1371/journal.pone.0240295)
Supplement: S1 File — (DOCX) [file pone.0240295.s001.docx]

Interview Questions

Gender Diverse Community Resilience

1. Just to start off, can you tell me about a time when you felt like you were connected to other gender diverse individuals? Can be recent or not. [Prompts: How did that feel?] –
2. Can you describe your connection to Transcend Charlotte, or lack of connection? (skip italics if not connected to Transcend)
3. How would you describe your social network? Not the specific people in it but when you look at it broadly.[Prompts: How many people would you say are in it? How do you mostly interact? Are they mostly friends, partners, family, work colleagues, etc?]
   1. In thinking specifically about other gender diverse folkx in your life, how would you think about that aspect of your social network? Is that any different?

**Transcend Questions**

1. *What makes you want to come to Transcend activities?*
2. What pulls you away/makes you not want to come to Transcend activities?
3. What are the effects on your daily life when you participate in gender diverse groups/services?
4. What are the effects on your daily life when you don’t participate in gender diverse groups/services?
5. What are some of the aspects that make Transcend MORE SUCCESSFUL as a community?
6. What are some of the aspects that DECREASE Transcend’s success in building a community?
   1. If we look at the group overall, what stressors is it facing do you think? (Prompts: other groups might face stresses like a lack of physical meeting space, or lack of leadership, these obviously don’t apply to Transcend but can you think of any of these type of group-level issues?)
7. Do you have another gender diverse group that you engage with? What makes that successful or not?
8. [If not addressed]: How are other identities like race/ethnicity, sexual identity, disability status, etc. included within Transcend? What about other gender diverse groups that you’re a part of?

**Personal resilience**

We’re going to ask some questions about resilience – here we mean about how you reduce the impact of stress or adversity on your life. You may or may not feel resilient all the time, and that’s pretty typical.

1. What are some of the major points of stress or types of adversity in your life?
2. How do you share resilience strategies with others in your network? [Prompts: There are ways that you have built strength, how do you let others know about some of the things you’ve figured out? Who do you mostly share that with? Do you only share that if they ask?]
   1. And how do you learn resilience strategies from others?

**Wrap up**

1. Confirm details from online survey.
   1. What name would you like to go by in the research? Can be real or fake.
   2. What pronouns would you like us to use if we are writing about this?
2. Are there any other questions that you thought I would ask you about that I haven’t?
