# Supplementary material for: “It just feels right”: Perceptions of the effects of community connectedness among trans individuals
Source: PLoS One. 2020 Oct 5;15(10):e0240295. doi: 10.1371/journal.pone.0240295 (PMC7535036; doi:10.1371/journal.pone.0240295)
Supplement: Attachment — Submitted filename: Response to reviewers.docx [file pone.0240295.s003.docx]

**Comments to the Author**

**Reviewer #2: Some great improvements on this paper. My remaining concerns are about the use of GD rather than trans or TGD as the overall population descriptor. GD seems to be slowly falling out of favour, and certainly in the realm of trans research overall, "trans"/"transgender" is the most appropriate title used when discussing these communities. I also noticed that "gender nonbinary" is still being used, which is generally a misnomer. "Non-binary" (without the word "gender" in front) is the most accepted gender descriptor in the majority of research in the area. So the first line of the abstract, for example, might read better as:
"Trans people (e.g. trans men and women, nonbinary and gender fluid people) are at higher risk for..."
Otherwise the paper is looking good, and once these little bits are fixed it should make for a good contribution.**

We appreciate the reviewer’s assistance with pointing out language changes and evolution. We have updated “gender diverse” to “trans” throughout the paper, and we have removed “gender” in front of “gender nonbinary” in the parenthetical descriptions.
